# Arsenic trioxide induces differentiation of CD133^+^ hepatocellular carcinoma cells and prolongs posthepatectomy survival by targeting *GLI1* expression in a mouse model

**DOI:** 10.1186/1756-8722-7-28

**Published:** 2014-03-30

**Authors:** Ke-Zhi Zhang, Qiang-Bo Zhang, Quan-Bao Zhang, Hui-Chuan Sun, Jian-Yang Ao, Zong-Tao Chai, Xiao-Dong Zhu, Lu Lu, Yuan-Yuan Zhang, Yang Bu, Ling-Qun Kong, Zhao-You Tang

**Affiliations:** 1 Liver Cancer Institute and Zhongshan Hospital, Fudan University, Key Laboratory for Carcinogenesis and Cancer Invasion, The Chinese Ministry of Education, 136 Yi Xue Yuan Road, Shanghai 200032, P R China; 2 Department of General Surgery, Qilu Hospital, Shandong University, Jinan 250012, P R China

**Keywords:** Arsenic trioxide, Hepatocellular carcinoma, Cancer stem cell, Differentiation, *GLI1*

## Abstract

**Background:**

Cancer stem cells (CSCs) play a key role in the posthepatectomy recurrence of hepatocellular carcinoma (HCC). CD133^+^ HCC cells exhibit liver CSC–like properties, and CSC differentiation–inducing therapy may lead these cells to lose their self-renewal ability and may induce terminal differentiation, which may in turn allow their malignant potential to be controlled. Because arsenic trioxide (As_2_O_3_) increases remission rates and prolongs survival among patients with acute promyelocytic leukemia by inducing differentiation and apoptosis of leukemic cells, we hypothesized that As_2_O_3_ might also inhibit HCC recurrence and prolong survival time after hepatectomy by inducing differentiation of HCC CSCs.

**Methods:**

We evaluated the As_2_O_3_ induced differentiation of human HCC CSCs and its mechanism *in vitro*, and we investigated the effects of treatment with As_2_O_3_ on recurrence rates and median survival in a mouse xenograft model.

**Results:**

We found that As_2_O_3_ induced HCC CSC differentiation by down-regulating the expression of CD133 and some stemness genes, thus inhibiting the cells’ self-renewal ability and tumorigenic capacity without inhibiting their proliferation *in vitro. In vivo* experiments indicated that As_2_O_3_ decreased recurrence rates after radical resection and prolonged survival in a mouse model. As_2_O_3_, which shows no apparent toxicity, may induce HCC CSC differentiation by down-regulating the expression of *GLI1*.

**Conclusions:**

We found that As_2_O_3_ induced HCC CSC differentiation, inhibited recurrence, and prolonged survival after hepatectomy by targeting *GLI1*expression. Our results suggest that the clinical safety and utility of As_2_O_3_ should be further evaluated.

## Background

Worldwide, hepatocellular carcinoma (HCC) is the fifth most common cancer and the second leading cause of cancer death in men, and the seventh most common cancer and the sixth leading cause of cancer death in women [1]. At present, surgical resection is the first choice for treatment of HCC, but the long-term prognosis remains unsatisfactory; the recurrence rate is high, owing to the lack of an effective adjuvant therapy [2]. Fan et al. reported that the presence of circulating liver cancer stem cells (CSCs) is closely related to HCC recurrence and metastasis after resection [3]. Recent studies have suggested that antiangiogenic therapy can stimulate tumor invasion and metastasis [4,5], and the mechanism of this stimulation may also be related to CSCs [6].

CSCs have the ability to self-renew, to differentiate into defined progeny, and, most importantly, to initiate and sustain tumor growth, and they play a key role in tumor progression, metastasis, and recurrence [7]. CSCs also show resistance to chemotherapeutics and radiation [8]. In HCC, CD133^+^ cells exhibit liver CSC–like properties, such as high clonogenicity, tumorigenicity, and resistance to radiation [9-13]. In addition to CD133, EpCAM [14], CD44 [13] and CD90 [15] have also been used as markers for the identification of HCC CSCs. Tang et al. reported that CD133 expression overlaps extensively with expression of EpCAM and CD44, which suggests that stem cells marked by CD133/EpCAM and possibly CD133/CD44 may have similar characteristics and regulatory mechanisms [11]. Tang et al. also showed that CD90 is expressed at very low levels in HCC cell lines and clinical specimens. Other studies have shown that the presence of CD133^+^ CSCs in HCC patients after surgery is correlated with early recurrence and poor prognosis [16,17]. Two studies have reported that hepatocyte nuclear factor 4 alpha and bone morphogenetic protein 4 can promote the differentiation of CD133^+^ HCC stem cells, inhibition of self-renewal, and resistance to chemotherapy [18,19]; and these results suggest that inducing CSC differentiation is a promising approach to the treatment of HCC. However, the use of differentiation-inducing drugs for HCC has not been well explored.

A number of studies have shown that the Hedgehog (HH) signaling pathway plays an important role in CSC self-renewal and inhibition of tumor differentiation in various cancers [20-23]. Targeting HH signaling has been shown to inhibit self-renewal of melanoma CSCs *in vitro* and to decrease their tumor initiation ability *in vivo*[24]. In a clinical study, advanced basal cell carcinoma patients were found to benefit from inhibition of HH signaling [25]. In HCC, *GLI1* expression is significantly correlated with tumor size and TNM stage; *GLI1* expression is high in patients with early recurrence and poorer overall survival [26]. Therefore, *GLI1* may be a useful target for the prevention of HCC recurrence after surgery.

Arsenic trioxide (As_2_O_3_) induces differentiation and apoptosis of acute promyelocytic leukemia (APL) cells by binding to the PML-RARα oncoprotein [27], and treatment with As_2_O_3_ has been shown to increase remission rates and prolong survival in patients [28]. Some studies have shown that As_2_O_3_ inhibits the growth and metastasis of HCC by inducing apoptosis [29,30], but a clinical trial showed that treatment with As_2_O_3_ is not effective for patients with advanced HCC [31]. There have been no reports of As_2_O_3_ preventing HCC recurrence or inducing differentiation of HCC CSCs.

We hypothesized that As_2_O_3_ could induce differentiation of CD133^+^ HCC cells through the HH-GLI pathway, and also investigated whether As_2_O_3_ could inhibit HCC recurrence and prolong survival time after hepatectomy in a mouse model.

## Results

### As_2_O_3_ reduced CD133 expression in HCC CSCs but did dot inhibit proliferation at lower dosages

**Figure 1:**
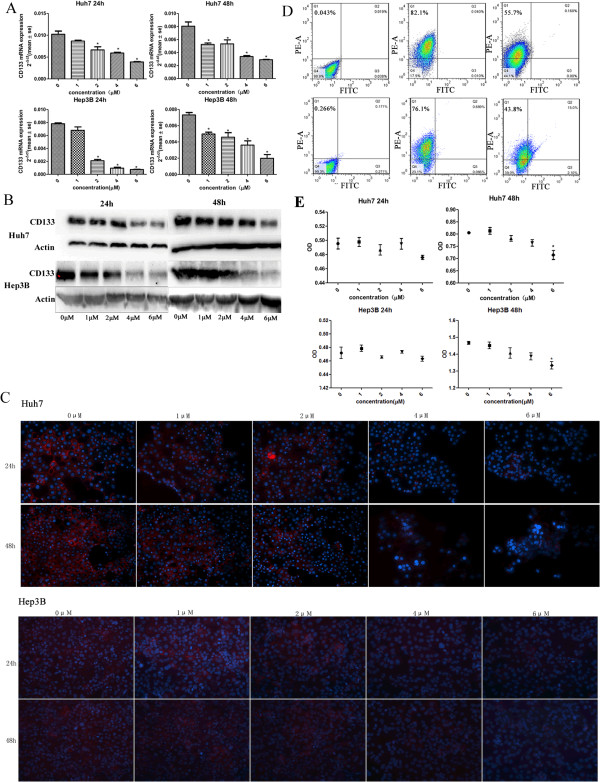
**As**_**2**_**O**_**3 **_**down-regulated CD133 expression *in vitro *and did not inhibit cell proliferation at lower dosages. (A–C)** Treatment of CD133^+^ HCC cells, with 2 μM As_2_O_3_ reduced CD133 mRNA expression after 24 hours and treatment with 1 μM As_2_O_3_ did so after 48 hours (**A**, *P < 0.05) ; treatment with As_2_O_3_ also reduced CD133 protein expression (**B,C**; red, CD133; blue, 4′,6-diamidino-2-phenylindole; magnification, ×200). **(D) After** treatment with 4 μM As_2_O_3_ for 5 days, the percentage of CD133^+^ Huh7-wt cells decreased from 82.1% to 55.7% (upper panels) and the percentage of CD133^+^ Hep3B-wt cells decreased from 76.1% to 43.8% (lower panels). **(E)** In a 48-hour assay, 6 μM As_2_O_3_ inhibited proliferation of HCC CSCs, whereas As_2_O_3_ at 1–4 μM had little effect on proliferation over the course of 24 or 48 hours (*P < 0.05).

We used unmodified human hepatoma cell lines (Huh7-wt and Hep3B-wt), which have a relatively high percentage of CD133^+^ cells, to analyze the biologic effects of As_2_O_3_ on HCC CSCs. Various dosages of As_2_O_3_ (0–6 μM) were used to explore its effects on CSC differentiation. We found that in CD133^+^ cells, As_2_O_3_ significantly reduced expression of CD133 mRNA and protein and that mRNA expression decreased with increasing As_2_O_3_ concentration (Figure 1A–C). Because 4 μM As_2_O_3_ significantly down-regulated CD133 expression, we used this concentration for subsequent tests. Flow cytometry results indicated that treatment with As_2_O_3_ decreased the percentage of CD133^+^ cells by more than 20% (Figure 1D).

To determine whether As_2_O_3_ inhibited proliferation of CD133^+^ HCC cells, we performed a CCK8 cell proliferation assay, the results of which showed that As_2_O_3_ had little effect on CD133^+^ cell proliferation at 1–4 μM (Figure 1E).

### As_2_O_3_ down-regulated expression of stemness-associated genes of HCC CSCs

**Figure 2:**
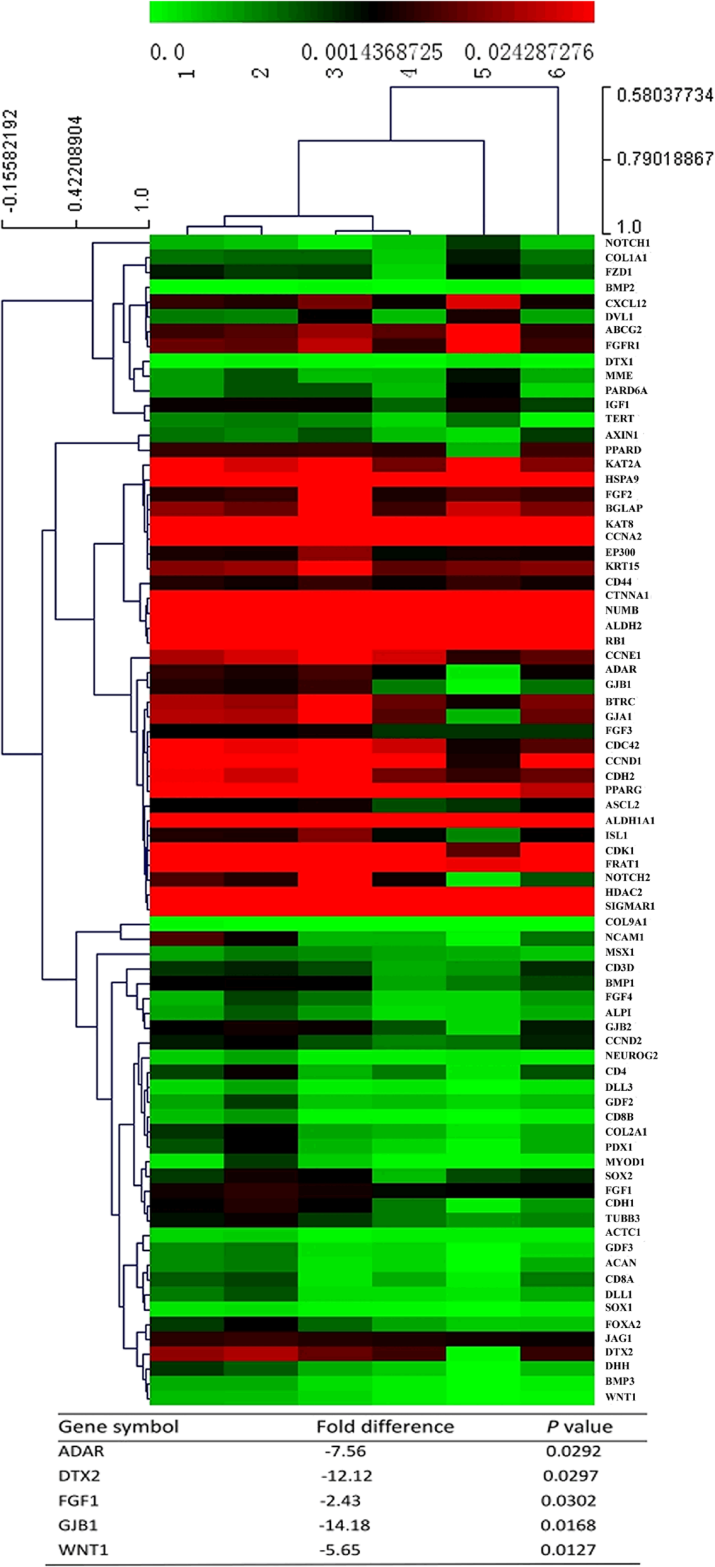
**As**_**2**_**O**_**3 **_**down-regulated expression of 5 stemness genes in HCC CSCs.**

To evaluate the alteration of stemness gene expression during the process by which As_2_O_3_ induced differentiation of HCC CSCs, we analyzed stemness gene expression by means of a Human Stem Cell RT^2^ Profiler PCR Array after the CD133^+^ Huh7-wt cells had been treated with 4 μM As_2_O_3_ for 48 hours. The data showed that reduced expression of CD133 after As_2_O_3_ treatment was accompanied by down-regulation of 5 stemness genes that are involved in the maintenance of pluripotency or are richly expressed in human CSCs [13,32-35]: adenosine deaminase, RNA-specific (*ADAR*); gap junction protein, beta 1, 32 kDa (*GJB1*); fibroblast growth factor 1 (acidic) (*FGF1*); deltex homolog 2 (Drosophila) (*DTX2*); and wingless-type MMTV integration site family, member 1 (*WNT1*) (Figure 2). These 5 stemness genes were also down-regulated in CD133^+^ Hep3B-wt cells that had been treated with 4 μM As_2_O_3_ for 48 hours, as indicated by real-time polymerase chain reaction (Additional file 1: Table S1).

### As_2_O_3_ inhibited self-renewal and tumorigenic capacity of HCC CSCs

**Figure 3:**
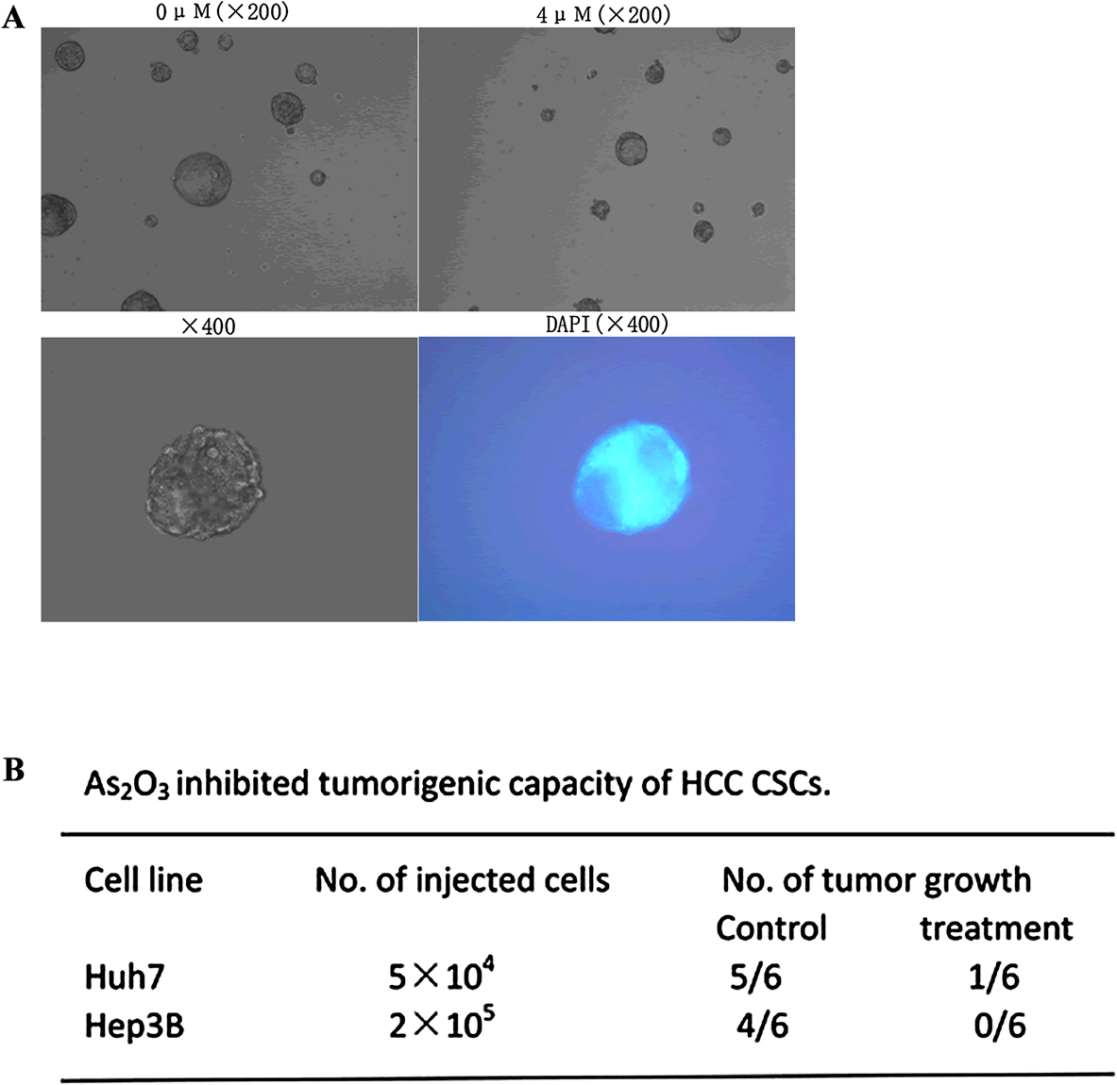
**As**_**2**_**O**_**3 **_**inhibited self-renewal and decreased the tumorigenic capacity of CD133**^**+ **^**CSCs. (A)** Pretreatment of CD133^+^ Huh7-wt and CD133^+^ Hep3B-wt cells with 4 μM As_2_O_3_ for 5 days inhibited CSC sphere formation. **(B)** As_2_O_3_ also decreased the tumorigenicity of CD133^+^ cells.

The fundamental properties of CSCs are their ability to self-renew and their ability to differentiate into defined progeny. To evaluate the self-renewal potential of CD133^+^ cells after treatment with As_2_O_3_*in vitro*, we used a sphere-forming assay. We tested the sphere-formation ability of CD133^+^ cells incubated with or without As_2_O_3_ for 5 days and found that tumor spheres generated from CD133^+^ cells treated with As_2_O_3_ were fewer in number and smaller in size than the spheres generated from untreated cells (Figure 3A).

To compare the tumorigenic potential of As_2_O_3_-treated and untreated CD133^+^ HCC cells, we inoculated NOD/SCID mice subcutaneously with 5 × 10^4^ CD133^+^ Huh7-wt cells or 2 × 10^5^ CD133^+^ Hep3B-wt cells, and we found that tumor growth was inhibited by As_2_O_3_ treatment (Figure 3B).

### As_2_O_3_ inhibited recurrence after curative resection and improved survival in a mouse model

The incidences of intrahepatic tumor recurrence in the control and As_2_O_3_-treated groups were 75% (9/12) and 33.33% (4/12) for the Huh7-GFP cell line and 91.67% (11/12) and 41.67% (5/12) for the Hep3B-GFP cell line, and the differences were statistically significant. No lung metastasis was observed in either group. No animal experienced a weight loss of more than 10%, fever, or anemia during the treatment regimen. We also found there were no significant pathological changes in the heart, spleen, or kidney of the mice in the As_2_O_3_ group.

**Figure 4:**
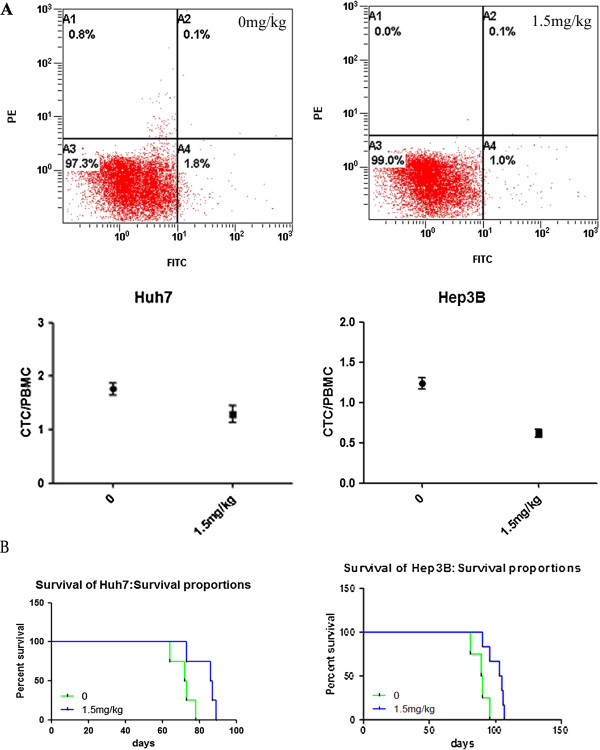
**As**_**2**_**O**_**3 **_**decreased the proportion of CTCs relative to peripheral blood mononuclear cells (PBMCs) and prolonged survival. (A)** Flow cytometry analysis indicated that As_2_O_3_ significantly decreased the proportion of CTCs relative to PBMCs, In the Huh7-GFP cell line, the proportions of CTCs relative to PBMCs were 1.292% ± 0.164% in the As_2_O_3_ group and 1.758% ± 0.118% in the control group (*P* = 0.0305), and in the Hep3B-GFP cell line, the corresponding proportions were 0.619% ± 0.050% and 1.235% ± 0.072% (*P* < 0.0001). **(B)** In the Huh7-wt cell line, median survival increased from 72.5 to 86.5 days (*P* = 0.0344), and in the Hep3B-wt cell line, median survival increased from 89.5 to 104 days (*P* = 0.015).

Recurrence after resection is closely related to the presence of circulating tumor cells (CTCs) in patients with HCC [36]. Therefore, we compared the numbers of CTCs in the As_2_O_3_ and control groups (Figure 4A) and found that As_2_O_3_ treatment significantly decreased the number of CTCs.

In both the Huh7-wt model and the Hep3B-wt model, treatment with As_2_O_3_ for 4 weeks significantly prolonged survival (Figure 4B).

### As_2_O_3_ down-regulated *GLI1* expression via the HH signaling pathway

**Figure 5:**
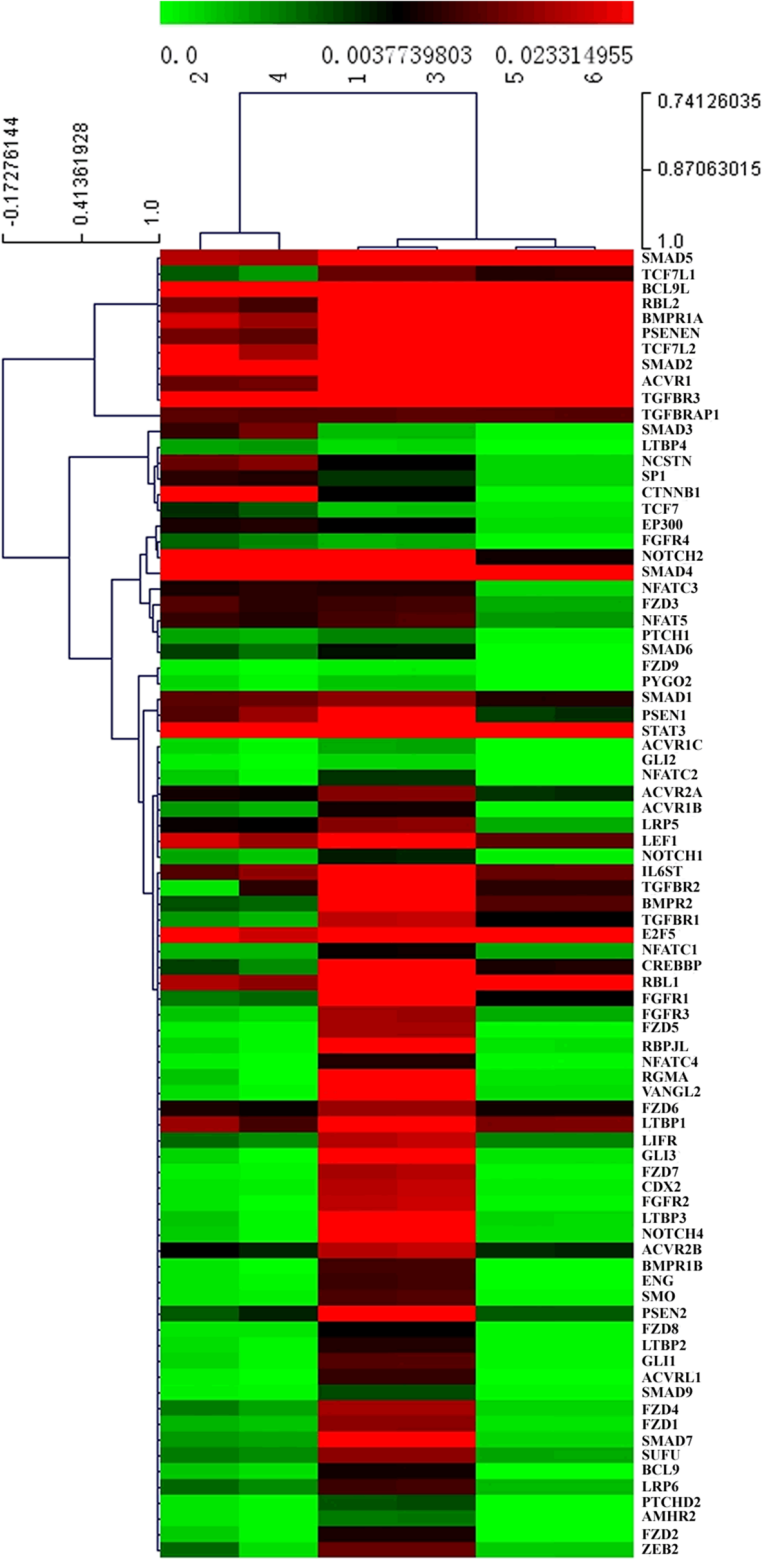
**The results of Human Stem Cell Signaling RT**^**2 **^**Profiler PCR Array analysis indicated that As**_**2**_**O**_**3 **_**down-regulated the expression of *GLI1 *** **and *PTCH1 *** **in CD133**^**+ **^**Huh7-wt cells.**

**Figure 6:**
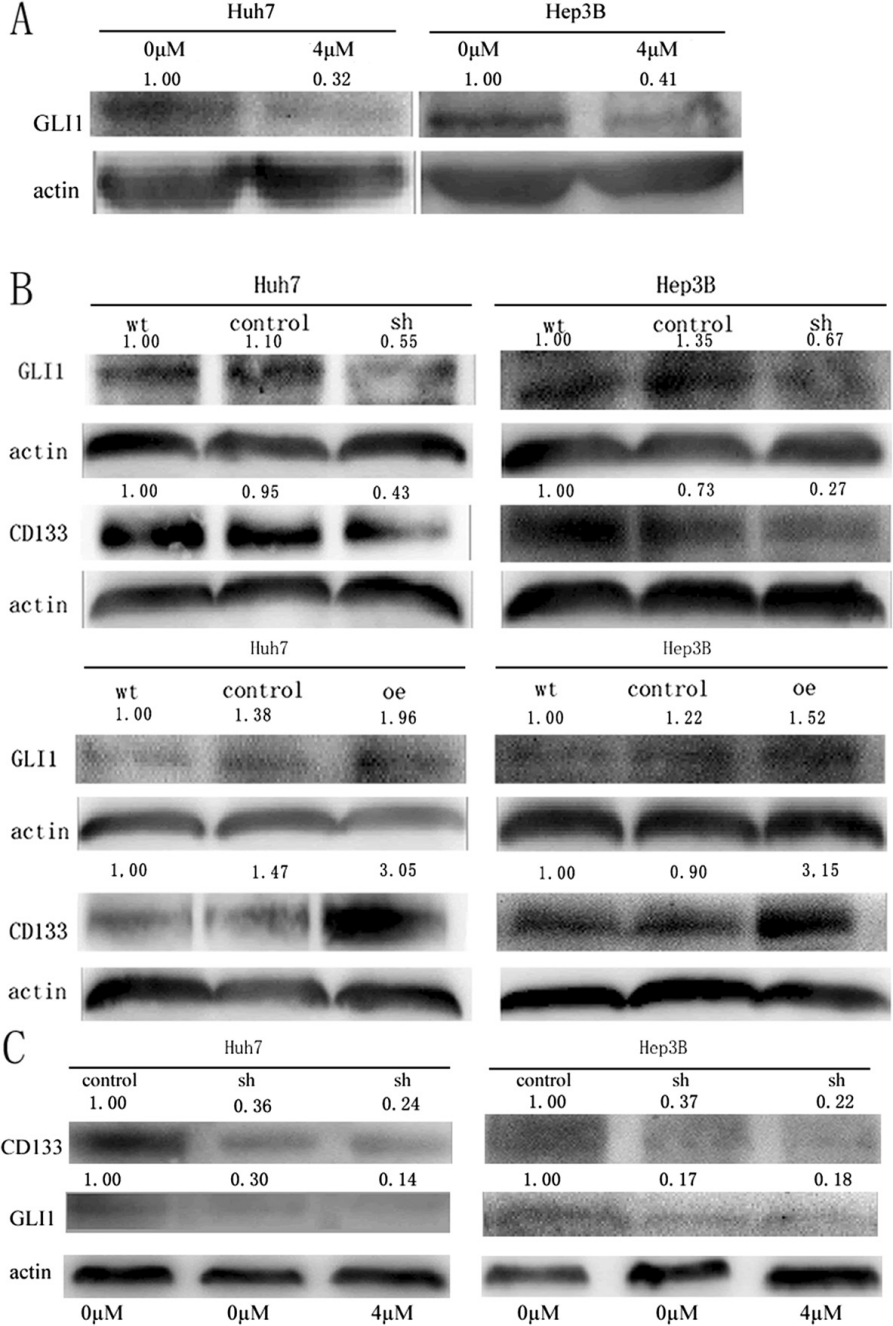
**As**_**2**_**O**_**3 **_**down-regulated *GLI1 *expression via the Hedgehog signaling pathway. (A)**, As_2_O_3_ decreased *GLI1* protein expression in CD133^+^ Huh7-wt and CD133^+^ Hep3B-wt cells, as indicated by Western blotting analysis. **(B)**, Short hairpin RNA knockdown of *GLI1* expression decreased CD133 expression in CD133^+^ Huh7-wt and CD133^+^ Hep3B-wt cells; similarly, up-regulation of *GLI1* expression increased CD133 expression (wt = CD133^+^ Huh7-wt and CD133^+^ Hep3B-wt; control = Huh7-LV-shNon, Hep3B-LV-shNon, and Huh7-LV-oeNon, Hep3B-LV-oeNon; sh = Huh7-LV-shGLI1 and Hep3B-LV-shGLI1; oe = Huh7-LV-oeGLI1 and Hep3B-LV-oeGLI1). **(C)**, Huh7-LV-shGLI1 and Hep3B-LV-shGLI1 cells were treated with 4 μM As_2_O_3_ for 48 hours, the expression of CD133 did not decrease significantly.

To explore how As_2_O_3_ induced differentiation of CD133^+^ HCC CSCs, we analyzed stem cell signaling–related gene expression by using a Human Stem Cell Signaling RT^2^ Profiler PCR Array after the CD133^+^ Huh7-wt cells had been treated with 4 μM As_2_O_3_ for 48 hours. We found that As_2_O_3_ treatment down-regulated expression of *GLI1* and *PTCH1* (−10.13 fold, *P* = 0.0279); (Figure 5). Combined with the results of the Human Stem Cell RT^2^ Profiler PCR Array, these results indicate that As_2_O_3_ down-regulated the expression of *GLI1* and its downstream target genes *PTCH1* and *WNT1*, so we hypothesized that As_2_O_3_ inhibited the expression of CD133 by down-regulating *GLI1*. This hypothesis was confirmed by Western blotting analysis in the CD133^+^ Huh7-wt and CD133^+^ Hep3B-wt models (Figure 6A). Furthermore, we found that knockdown of *GLI1* expression or forced over-expression of *GLI1* significantly decreased or increased, respectively, CD133 expression (Figure 6B). Huh7-LV-shGLI1 and Hep3B-LV-shGLI1 cells were treated with 4 μM As_2_O_3_ for 48 hours, the expression of CD133 did not decrease significantly (Figure 6C), and this results support the notion that As_2_O_3_ may decrease CD133 expression by targeting *GLI1*.

## Discussion

The CSC theory is based on the fact that CSCs exhibit properties similar to those of normal stem cells, such as self-renewal, the ability to produce heterogeneous progeny, and the ability to divide in an unlimited way, giving rise to high tumorigenicity, chemotherapy and radiation resistance, metastasis, and cancer recurrence after therapy [8]. Therefore, CSCs have the potential to be effective targets for preventing relapse after resection, including in HCC. Therapy involving induced differentiation of CSCs could lead to the cells losing their self-renewal ability and could induce terminal differentiation. To date, the best example of the clinical use of differentiation-inducing therapy is treatment of APL with all-trans retinoic acid, which enhances the effects of chemotherapy and significantly improves patient outcome [37]. Targeting induction of CSC differentiation may be a powerful therapy for HCC. CD133^+^ HCC cells are considered to be CSCs because of their higher tumorigenicity [10] and lower expression of mRNAs for mature hepatocyte markers [9]. As_2_O_3_, which is an FDA-approved agent for the treatment of APL, can induce APL cells differentiation. Our results revealed that CD133 expression and the percentage of CD133^+^ cells were dramatically decreased in both CD133^+^ Huh7-wt and cd133^+^ Hep3B-wt cells treated with As_2_O_3_. But real-time PCR analysis revealed that after the CSCs were treated with As_2_O_3_ for 48 hours, a cluster of hepatocyte marker genes—including phosphoenolpyruvate carboxykinase, glucose-6-phosphatase, cytochrome P450, glutamine synthetase, biliverdin reductase, aldolase B, apolipoprotein c III, apolipoprotein A I, 4-hydroxyphenylpyruvate dioxygenase, and glycogen synthetase 2—were not re-expressed (data not shown). Furthermore, we found that As_2_O_3_ treatment decreased the expression of some stemness genes, which are important for the maintenance of CSC self-renewal ability and tumorigenicity [13,38]. We also found that high-dose As_2_O_3_ reduced CSC proliferation, whereas low-dose As_2_O_3_ had no such effect. CSCs are considered to be more resistant to chemotherapeutic agents than non-CSCs, and consistent with this, low-dose As_2_O_3_ has been found to inhibit Huh7-wt cell proliferation [39]. In short, low-dose As_2_O_3_ may induce HCC CSCs differentiation into non-CSCs,which is very important for the clinical application of As_2_O_3_ because of its toxicity.

It should be possible to treat cancer by differentiation-inducing therapy targeted at CSCs. If CSCs could be induced to differentiate, then their malignant potential could be controlled [34]. We have shown that As_2_O_3_ induced HCC CSC differentiation *in vitro*, and thus we postulate that As_2_O_3_ may be useful for inhibiting recurrence of HCC after resection and may prolong survival *in vivo*. The HCC recurrence rate was decreased by systemic administration of As_2_O_3_, and survival time was significantly prolonged. However, in a phase II clinical trial, patients with advanced HCC did not benefit from As_2_O_3_ treatment [31], and we believe that the effective treatment of advanced HCC must involve a combination of differentiation-inducing therapy and conventional chemotherapies to eradicate the tumor mass, and this belief is partly supported by the results of Wang et al. [13]. Due to the toxic effects of As_2_O_3_ limits its clinical application, we found that low-dosage of As_2_O_3_ can induce differentiation of HCC CSCs and inhibit recurrence, so the application of As_2_O_3_ to prevent recurrence of HCC patients after surgery may be an effective way. We also found CTCs were decreased by As_2_O_3_ treatment. Because the presence of CTCs is associated with metastasis to distant organs and is strongly associated with poor overall survival [40], we suggest that As_2_O_3_ could decrease metastasis and prolong survival. This suggestion could be tested with other models.

The HH-GLI pathway is an important mechanism for determining embryonic pattern and regulating cell fate [11]. This pathway has been implicated in several types of tumors [41] and plays an important role in the differentiation of CSCs [20-23]. Differentiation of CSCs by inhibition of the HH-GLI pathway may be a promising therapeutic strategy for human tumors. Currently, all the therapeutics in clinical development that function by inhibiting the HH-GLI pathway are targeted at *SMO*[41]. However, in some types of tumors, specifically those in which increased *GLI* expression or activation is induced in a *SMO*-independent manner, *SMO* inhibitors are ineffective, and inhibitors that modulate *GLI* may be useful [42-44]. In HCC, expression of *GLI1* mRNA has been reported to adversely affect recurrence and survival of patients with HCC after resection [26], which is consistent with our results, As_2_O_3_ may induce CSC differentiation by down-regulating the expression of *GLI1*, thus inhibiting recurrence and prolonging survival.

## Conclusion

In conclusion, As_2_O_3_ may induce HCC CSC differentiation, inhibit cancer recurrence after radical resection, and prolong survival as a result of down-regulation of *GLI1* expression. In *in vivo* tests, As_2_O_3_ shows no apparent toxicity, but its clinical safety and utility must be evaluated.

## Methods

### Cell culture and reagents

Unmodified Huh7 and Hep3B cells (Huh7-wt and Hep3B-wt) were obtained from American Type Culture Collection. Cells from both cell lines were transfected with GFP (Huh7-GFP and Hep3B-GFP, respectively) as described previously [45]. Huh7-LV-shGLI1 and Hep3B-LV-shGLI1 cells were obtained by infecting CD133^+^ Huh7-wt and CD133^+^ Hep3B-wt with a lentiviral vector encoding short hairpin RNA for *GLI1* (sc-37911-V, Santa Cruz Biotechnology, Santa Cruz, CA, USA) to silence its expression. As controls, Huh7-LV-shNon and Hep3B-LV-shNon cells were obtained by infection of CD133^+^ Huh7-wt and CD133^+^ Hep3B-wt with a different lentiviral vector (sc-108080, Santa Cruz Biotechnology). Huh7-LV-oeGLI1 and Hep3B-LV-oeGLI cells were obtained by infecting CD133^+^ Huh7-wt and CD133^+^ Hep3B-wt with lentiviral vectors that overexpress *GLI1* (Genechem, Shanghai, P.R. China). Huh7-LV-oeNon and Hep3B-LV-oeNon cells were also used as controls (Genechem). All cell lines were cultured in Dulbecco’s modified Eagle’s medium supplemented with 10% fetal bovine serum.

As_2_O_3_ (SL Pharm, Beijing, P.R. China) was dissolved at a concentration of 0.05 mg/ml in phosphate buffered saline (PBS) for the *in vitro* study. For the *in vivo* study, As_2_O_3_ was dissolved in normal saline.

### Cell isolation by fluorescence-activated cell sorting

Huh7-wt and Hep3B-wt cells were labeled directly with phycoerythrin-conjugated anti-human CD133/1 antibody (Miltenyi Biotec, Gladbach, Germany) according to the manufacturer’s instruction and were sorted by fluorescence-activated cell sorting to obtain CD133^+^ cell subpopulations; a sorted cell purity exceeding 90% was deemed acceptable for *in vitro* experiments.

### Cell proliferation assay

Cell proliferation was counted by a CCK8 assay (Dojindo, Tokyo, Japan). Three thousand CD133^+^ cells were seeded in 96-well culture plates. After adhesion for 24 hours, the cells were treated with As_2_O_3_ at a final concentration ranging from 1 to 6 μM, and the treated cells were cultured for another 24 or 48 hours. Cells that were not exposed to As_2_O_3_ were used as controls. The cell proliferation assay was carried out as previously described [5]. The optical density values were read by an enzyme-linked immunosorbent assay reader at 450 nM.

### Real-time polymerase chain reaction analysis

Real-time PCR analysis was carried out as described as elsewhere [5]. The following primers were used for amplification of human genes: CD133 [18], forward 5′- ACATGAAAAGACCTGGGGG-3′, reverse 5′- GATCTGGTGT CCCAGCATG-3′; β-actin, forward 5′-GCTCTGCAGACTTCAGACCA-3′, reverse 5′-GGCCGGACTCATCGTACTCCTGC-3′.

### Western blotting assay

The procedure used for Western blotting assay is described elsewhere [5]. Primary antibodies included anti-CD133/1 (Miltenyi Biotec), anti-GLI1 (Santa Cruz Biotechnology), and anti-β-actin (Kangcheng Technology, Shanghai, P.R. China).

### Immunofluorescence

To assess the distribution and changes of CD133 in HCC CSCs after treatment with As_2_O_3_, we stained CD133^+^ Huh7-wt and CD133^+^ Hep3B-wt cells for visualization by means of an immunofluorescence assay. Briefly, the cells were seeded on slides, allowed to adhere for 24 hours, treated with As_2_O_3_ at a final concentration ranging from 1 to 6 μM, and then cultured for another 24 or 48 hours. Cells that were not exposed to As_2_O_3_ were used as controls. The slides were washed with PBS and fixed with 4% paraformaldehyde. The cells were incubated with primary antibody to CD133/1 (Miltenyi Biotec) overnight at 4°C after being blocked with 5% bovine serum albumin, and then goat anti-mouse tetramethyl rhodamine isothiocyanate-conjugated secondary antibody was added before staining with 4′,6-diamidino-2-phenylindole. Fluorescence images were visualized with an inverted fluorescence microscope (Olympus, Melville, NY, USA). For a negative control, primary antibodies were replaced with PBS.

### Flow cytometry analysis

The percentages of CD133^+^ cells were determined by flow cytometry to evaluate the expression of CD133. Isolated CD133^+^ HCC cells were incubated with or without As_2_O_3_ for 5 days. The cells were trypsinized, washed, and resuspended in PBS. Then the cells were incubated with phycoerythrin-conjugated anti-CD133/1 antibody (Miltenyi Biotec) on ice for 30 min and analyzed by means of flow cytometry (BD Biosciences, USA).

### Culture of HCC CSC spheres

The self-renewal capability of HCC cells was evaluated by testing their sphere-formation ability. Specifically isolated CD133^+^ HCC cells treated with 4 μM As_2_O_3_ treatment for 5 days and untreated cells were cultured in a methyl cellulose–based medium in low-adherent 96-well culture plates (Corning, Corning, NY, USA) under serum-free conditions and supplemented with 20 μg/ml insulin, 20 μg/ml epidermal growth factor, and 10 μg/ml basic fibroblast growth factor (RD Systems, Minneapolis, MN, USA) according to a previously published procedure [46]. Single-cell suspensions of 100 CD133^+^ HCC cells were seeded, and epidermal growth factor, basic fibroblast growth factor, and insulin were added every second day for 2 weeks.

### Tumorigenic capacity of HCC CSCs

The tumorigenic capacity of 4 μM As_2_O_3_-treated and untreated CD133^+^ HCC cells for 5 days were analyzed in an NOD/SCID mouse xenograft model. Male NOD/SCID mice (4–6 weeks old) were injected subcutaneously in the lateral flanks with 5 × 10^4^ CD133^+^ Huh7-wt cells and 2 × 10^5^ CD133^+^ Hep3B-wt cells. Tumorigenic capacity was evaluated after the cells had been allowed to undergo implantation for 7 weeks.

### Animal model

Male BALB/c nu/nu nude mice (Shanghai Institute of Materia Medica, Chinese Academy of Science, Shanghai, P.R. China) were housed in laminar-flow cabinets under specific pathogen-free conditions and used when they weighed approximately 20 g and were 4–6 weeks old. The experimental protocol was approved by the Shanghai Medical Experimental Animal Care Committee. The animal model was established as previously described [47]. Briefly, Huh7-wt, Hep3B-wt, Huh7-GFP, and Hep3B-GFP cells (1 × 10^7^) were subcutaneously inoculated into the right flanks of the nude mice. After 3–4 weeks, a piece of non-necrotic tumor tissue about 2 mm in size was orthotopically implanted into the liver. On the fourteenth day after implantation, the tumor was excised, with the length between incisional margin and tumor edge being ≥ 2 mm.

### As_2_O_3_ treatment and grouping

The *in vivo* experiment involved 2 groups: a recurrence rate group, which used the Huh7-GFP and Hep3B-GFP cell lines, and a survival observation group, which used the Huh7-wt and Hep3B-wt cell lines. In the recurrence rate group, treatment was started on the fifth day after resection. The mice were randomly assigned to 2 groups (*n* = 12 for each group): one group received a daily intraperitoneal dose of 1.5 mg/kg As_2_O_3_, and the other group received an equal volume of normal saline (control group). The treatment was continued for 2 weeks, and after another 3 weeks, the mice were anesthetized and orbital blood was obtained. The mice were then sacrificed by cervical dislocation to determine the recurrence rate.

In the survival group, treatment was started on the fifth day after resection. The mice were randomly assigned to 2 groups (*n* = 8 for each group): one group received a daily intraperitoneal dose of 1.5 mg/kg As_2_O_3_, and the other group received an equal volume of normal saline (control group). The treatment was administered for 2 weeks, then stopped for 1 week to allow the mice to recover, and then administered for an additional 2 weeks; then the median survival was determined.

### Detection of CTCs

CTCs in the peripheral blood of mice in the recurrence group were counted by flow cytometry, with GFP as a marker. The protocol was carried out as previously reported [15]. The GFP-positive cells were gated with processed blood from a mouse that had not been subjected to the xenograft procedure.

### PCR microarray analysis of stemness gene expression and pathway exploration

Total RNA was extracted from isolated As_2_O_3_-treated and untreated CD133^+^ HCC cells and was analyzed by means of a Human Stem Cell RT^2^ Profiler PCR Array ( http://www.sabiosciences.com/rt_pcr_product/HTML/PAHS-405Z.html) and a Human Stem Cell Signaling RT^2^ Profiler PCR Array ( http://www.sabiosciences.com/rt_pcr_product/HTML/PAHS-047Z.html), according to the manufacturer’s instructions.

### Statistical analysis

Values for continuous variables are expressed as means ± SDs and were compared by means of the unpaired 2-tailed Student *t* test, unless otherwise specified, Multiple comparisons of Kaplan-Meier curves were made using the log-rank test. All statistical tests were performed using SPSS for Windows (ver. 120.0, SPSS, Inc). *P* < 0.05 (2-tailed) was considered to indicate statistical significance.

## Competing interests

The authors declare that they have no competing interests.

## Authors’ contributions

ZKZ designed the study, established the animal model, carried out the Western blotting assays, performed the statistical analysis, and drafted the manuscript. ZQB and ZQB participated in the design of the study, data analysis, and drafting the manuscript. SHC participated in the design of the study and helped to draft the manuscript. AJY, CZT, ZXD, LL, ZYY, BY, and KLQ helped to acquire experimental data. TZY conceived the study, participated in its design and coordination, and helped to draft the manuscript. All authors read and approved the final manuscript.

## Supplementary Material

Additional file 1: Table S1 Real-time PCR primers for the 5 stemness genes that were down-regulated in CD133^+^ Hep3B-wt cells after treatment with As_2_O_3_ for 48 hours. Click here for file
