# Supplementary material for: Arsenic trioxide induces differentiation of CD133+ hepatocellular carcinoma cells and prolongs posthepatectomy survival by targeting GLI1 expression in a mouse model
Source: J Hematol Oncol. 2014 Mar 30;7:28. doi: 10.1186/1756-8722-7-28 (PMC4022144; doi:10.1186/1756-8722-7-28)
Supplement: Additional file 1: Table S1 — Real-time PCR primers for the 5 stemness genes that were down-regulated in CD133+ Hep3B-wt cells after treatment with As2O3 for 48 hours. [file 1756-8722-7-28-S1.docx]

**Additional file 1: Table S1 5 stemness genes real-time PCR primers used in this study**

**Homo sapiens gene Forward prime(5'- 3') Reverse prime(5' - 3')**

ADAR TTTCCCTCCAAACTGCTGAC GCTGCCTTCTGATGCTGAG

DTX2 GCCAGTGCTACCTTCCAGAC AACAAACCTCCCAGCCTCTC

FGF1 ACAAGGGACAGGAGCGAC TCCAGCCTTTCCAGGAACA

GJB1 AGAGGCACAAGGTCCACATCT AGCCATCCTGCTCACTCAG

WNT1 AGGTCTGAGGAGCAGCTTCA ATTGTCCACGCTGGATTTTC

**5 Stemness genes also downregulated in CD133^+^ Hep3B cells.**
